# IL-17, synoviolin and rheumatoid arthritis chronicity

**DOI:** 10.1186/ar3579

**Published:** 2012-02-09

**Authors:** Pierre Miossec

**Affiliations:** 1 Department of Immunology and Rheumatology, Hôpital Edouard Herriot, University of Lyon, France

## Background

The use of cytokine inhibitors has been a major progress in the treatment of chronic inflammation. However, not all patients respond and response will be often lost when treatment is stopped. These clinical aspects indicate that other cytokines might be involved and we focus here on the role of IL-17. In addition, the chronic nature of joint inflammation may contribute to reduced response and enhanced chronicity. We had previously observed that patients not responding well to TNF inhibition had higher blood expression of synoviolin, an E3 ubiquitin ligase previously shown to be implicated in synovial hyperplasia in human and mouse rheumatoid arthritis (RA). Therefore we studied the capacity of IL-17 to regulate synoviolin in human RA synoviocytes and in chronic reactivated streptococcal cell wall (SCW)-induced arthritis.

## Materials and methods

Chronic reactivated SCW-induced arthritis was examined in IL-17R deficient and wild-type mice. Synoviolin expression was analysed by real-time RT-PCR, Western Blot or immunostaining in RA synoviocytes and tissue, and p53 assessed by Western Blot. Apoptosis was detected by annexin V/ propidium iodide staining, SS DNA apoptosis ELISA kit or TUNEL staining and proliferation by PCNA staining. IL-17 receptor A (IL-17RA), IL-17 receptor C (IL-17-RC) or synoviolin inhibition were achieved by small interfering RNA (siRNA) or neutralizing antibodies.

## Results

IL-17 induced sustained synoviolin expression in RA synoviocytes. Sodium nitroprusside (SNP)-induced RA synoviocyte apoptosis was associated with reduced synoviolin expression and was rescued by IL-17 treatment with a corresponding increase in synoviolin expression. IL-17RC or IL-17RA RNA interference increased SNP-induced apoptosis, and decreased IL-17-induced synoviolin. IL-17 rescued RA synoviocytes from apoptosis induced by synoviolin knockdown. IL-17 and TNF had additive effects on synoviolin expression and protection against apoptosis induced by synoviolin knowndown. In IL-17R deficient mice, a decrease in arthritis severity was characterized by increased synovial apoptosis, reduced proliferation and a marked reduction in synoviolin expression. A distinct absence of synoviolin expressing germinal centres in IL-17R deficient mice contrasted with synoviolin positive B cells and Th17 cells in synovial germinal centre-like structures.

## Conclusions

IL-17 induction of synoviolin may contribute in part to RA chronicity by prolonging the survival of RA synoviocytes and immune cells in germinal centre reactions. These results extend the role of IL-17 to synovial hyperplasia.

